# *Fog2* Is Required for Normal Diaphragm and Lung Development in Mice and Humans

**DOI:** 10.1371/journal.pgen.0010010

**Published:** 2005-06-17

**Authors:** Kate G Ackerman, Bruce J Herron, Sara O Vargas, Hailu Huang, Sergei G Tevosian, Lazaros Kochilas, Cherie Rao, Barbara R Pober, Randal P Babiuk, Jonathan A Epstein, John J Greer, David R Beier

**Affiliations:** 1 Division of Emergency Medicine, Department of Medicine, Children's Hospital and Harvard Medical School, Boston, Massachusetts, United States of America; 2 Divison of Genetics, Brigham and Women's Hospital and Harvard Medical School, Boston, Massachusetts, United States of America; 3 Genomics Institute, Wadsworth Center, Troy, New York, United States of America; 4 Department of Pathology, Children's Hospital and Harvard Medical School, Boston, Massachusetts, United States of America; 5 Department of Genetics, Dartmouth Medical School, Hanover, New Hampshire, United States of America; 6 Department of Pediatrics, Brown Medical School, Providence, Rhode Island, United States of America; 7 Department of Surgery, Children's Hospital and Harvard Medical School, Boston, Massachusetts, United States of America; 8 Department of Physiology, University of Alberta, Edmonton, Alberta, Canada; 9 Cardiovascular Division, Department of Medicine and Department of Cell and Molecular Biology, University of Pennsylvania Medical School, Philadelphia, Pennsylvania, United States of America; Jackson Laboratory, United States of America

## Abstract

Congenital diaphragmatic hernia and other congenital diaphragmatic defects are associated with significant mortality and morbidity in neonates; however, the molecular basis of these developmental anomalies is unknown. In an analysis of E18.5 embryos derived from mice treated with N-ethyl-N-nitrosourea, we identified a mutation that causes pulmonary hypoplasia and abnormal diaphragmatic development. *Fog2 (Zfpm2)* maps within the recombinant interval carrying the N-ethyl-N-nitrosourea-induced mutation, and DNA sequencing of *Fog2* identified a mutation in a splice donor site that generates an abnormal transcript encoding a truncated protein. Human autopsy cases with diaphragmatic defect and pulmonary hypoplasia were evaluated for mutations in *FOG2*. Sequence analysis revealed a de novo mutation resulting in a premature stop codon in a child who died on the first day of life secondary to severe bilateral pulmonary hypoplasia and an abnormally muscularized diaphragm. Using a phenotype-driven approach, we have established that *Fog2* is required for normal diaphragm and lung development, a role that has not been previously appreciated. *FOG2* is the first gene implicated in the pathogenesis of nonsyndromic human congenital diaphragmatic defects, and its necessity for pulmonary development validates the hypothesis that neonates with congenital diaphragmatic hernia may also have primary pulmonary developmental abnormalities.

## Synopsis

Birth defects involving the diaphragm are as common and as serious as genetic disorders such as cystic fibrosis, yet the underlying causes of these defects are unknown. Most babies born with diaphragmatic defects have very small lungs, and many die in the newborn period with severe breathing difficulties. It is unknown whether the small lungs occur because these children have a diaphragmatic defect, or whether some patients might have a genetic abnormality that affects the development of both the lung and the diaphragm simultaneously.

In a screen of fetal mice carrying chemically induced genetic mutations, the authors found that a mutation in the gene *Fog2* *(Friend of gata 2),* causes abnormal diaphragm development and small lungs. The lungs have a primary developmental abnormality that includes the specific loss of one lung lobe. Based on this result, the authors studied children affected with diaphragmatic abnormalities, and identified one human baby with a serious mutation in the human gene *FOG2* who died at five hours of life with severe breathing difficulties, a diaphragmatic defect, and small lungs.

The authors have established that *Fog2* is necessary for both diaphragm and lung development in mice and in humans. This is the first known cause of a nonsyndromic congenital diaphragmatic defect and establishes that some patients may have a primary developmental abnormality of both the lung and the diaphragm.

## Introduction

Congenital diaphragmatic defects are a spectrum of relatively common birth defects. The Bochdalek or posterolateral hernias (often referred to as congenital diaphragmatic hernia [CDH]) occur in 1/3,000 live births [1], and although these are the most common type of diaphragmatic defect presenting at birth, diaphragmatic aplasia and diaphragmatic muscularization defects (eventrations) may have a similar clinical presentation.

Making specific anatomic distinctions among these types of defects can be difficult without direct gross (intraoperative or postmortem) examination. Pulmonary hypoplasia associated with these diaphragmatic defects causes severe mortality and morbidity. The pathogenesis and developmental relationship between diaphragmatic defects and pulmonary hypoplasia is not understood. Although advances in the medical management of pulmonary hypoplasia may have decreased the mortality associated with CDH patients who survive to receive care at high-volume centers [2,3], the population-based mortality has been reported to be as great as 62%, and there are a large number of deaths prior to birth or to transfer to a tertiary care facility [4]. As these patients commonly present with severe respiratory failure at birth, therapy has been centered around developing better methods to provide ventilatory support while not producing further lung injury. Extracorporeal membrane oxygenation (ECMO) is used in some centers to provide an extended period of cardiopulmonary bypass [5,6], while other centers have success using other ventilatory support techniques [7]. The morbidity in those who survive is high, and many patients survive with chronic respiratory insufficiency, cognitive and neuromotor deficits, and hearing loss as a result of necessary intensive interventions and associated structural and irreversible developmental abnormalities [8–11].

To date, there have been no specific mutations found to be associated with nonsyndromic diaphragmatic defects and pulmonary hypoplasia in humans. The heritability of these defects is unclear, as the high morbidity and mortality limit the collection of multigenerational families for analysis. The genetic etiologies are likely to be complex and probably arise from different mutations in various parts of the molecular developmental pathways required for diaphragmatic development. Indeed, there are numerous reports implicating different chromosomal abnormalities in the pathogenesis of CDH [12,13]. Given the difficulty of studying lethal developmental abnormalities in humans, it is of great potential utility to develop animal models of human birth defects, as the specific genetic abnormalities found in animal models can then be investigated in human populations.

We screened mice treated with the chemical mutagen N-ethyl-N-nitrosourea (ENU) for lines with developmental defects that present in the perinatal period [14]. From this screen, we identified a line of mice carrying a recessive mutation that results in primary pulmonary hypoplasia and abnormal diaphragmatic and cardiac development. Positional cloning analysis identified *Fog2 (Zfpm2)* as a likely candidate, and DNA sequencing revealed a mutation in a splice donor site that generates an abnormal transcript encoding a truncated protein. This result suggested that we examine the orthologous gene in humans with similar developmental defects, and we report the finding of a de novo nonsense mutation in *FOG2* in a patient who died at birth with a diaphragmatic defect and severe pulmonary hypoplasia. This is the first reported mutation associated with these abnormalities in a human. We present additional data that provide direct evidence that pulmonary hypoplasia may be a primary component of this spectrum of disorders.

## Results

### Identification of the *little lung* Mutation in *Fog2*

We screened third-generation progeny of ENU-mutagenized mice at embryonic day 18.5 (E18.5) for abnormal developmental phenotypes [14]. One line of mice was found to have multiple embryos in independent litters that displayed pulmonary hypoplasia and a thin diaphragm. The mutation, which we called *little lung* *(lil),* was mapped to Chromosome 15 by utilizing a strategy of interval haplotype analysis (data not shown) [15]. For high-resolution mapping, F2 progeny from two crosses were analyzed. In 450 progeny of an intercross of F1 (A/J × FVB/N) *lil/*+ mice, the interval containing the mutation was defined by 19 recombinants between *D15Mit220* and *D15Mit154*. Because of the lack of informative markers within this interval, an additional 39 F2 progeny from an A/J × C57BL/6J cross were tested. The identification of two recombinants established *D15Mit85* as the proximal and *D15Mit5* as the distal flanking markers.

**Figure 1:**
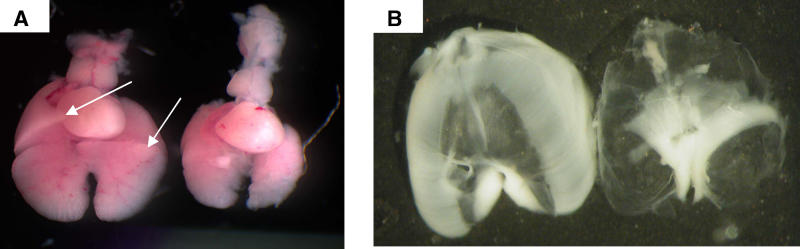
Abnormal Pulmonary and Diaphragmatic Development in the *lil* Mouse (A) The mutant hypoplastic lung (right) lacks the development of the accessory lobe and the anterior portion of the right middle lobe (marked with arrows on the control sample on the left). (B) Whole diaphragms show a lack of normal muscularization in the posterolateral regions and the peripheral regions of the mutant diaphragm (control on left and *lil* diaphragm on the right).

The *lil* phenotype was identified at E18.5 to have bilateral pulmonary hypoplasia and an abnormal diaphragm. Pulmonary hypoplasia was apparent in all mutant mice that survived to birth. In a comparison between wild-type and mutant mice found dead on day one of life, body weights were not different; however, the lung weights were significantly lower in the mutant mice. The average mutant lung weight was 9.6 ± 2.5 mg while the average wild-type lung weight was 26.4 ± 4.6 mg (*p* < 0.001). All lungs from mutant mice were lacking an accessory lobe on the right side and had underdevelopment of the anterior right middle lobe (Figure 1A). Diaphragms from mutant *lil* mice were intact, but muscularization was absent in the dorsal regions. Myotubules were present in a limited and abnormal distribution. More specifically, myotubules normally radiate in a mediolateral fashion to meet the lateral body walls, with a normal paucity of muscle fibers in the central tendonous region. In the mutant diaphragm, myotubules radiated in a dorsal–ventral orientation, and muscle tissue did not meet the entire surface of the body walls (Figure 1B).

The number of *lil* mutant mice that survived to birth was less than 5% of total progeny, rather than the 25% expected for a recessive mutation. We evaluated litters at different embryonic time points to determine whether the reduced number was due to intra-uterine demise. Litters were collected at E12.5, 13.5, 15.5, 17.5, and 18.5, and embryos were genotyped and evaluated for evidence of intra-uterine demise, including growth retardation, pallor, and tissue friability. *lil* embryos had a progressively higher rate of demise between E13.5 and E15.5. At E12.5, 20 out of 87 (23%) were homozygous for the mutation, and all of these appeared viable. At E13.5, 13 of 49 (27%) were mutant and two had died. At E15.5, 22 out of 91 (24%) were mutant and the majority of mutant embryos (17 out of the 22) had died. By E18.5, nine of 29 embryos (31%) were homozygous for the mutation, of which only one embryo was viable. Diaphragmatic muscularization was abnormal in all mutant mice examined (*n* > 25). Pulmonary hypoplasia was observed in 100% of mutants evaluated for that phenotype between E11.5 and birth (*n* > 50).

Examination of cardiac morphology showed that hearts from E15.5 *lil* mutant mice had a variety of developmental defects, including enlarged and abnormally developed endocardial cushions, a double-outlet right ventricle, and a complete atrioventricular canal. The myocardium was also poorly developed, with thinning of the outer compact layer and decreased vascularity. The cohort of mutant mice that survived to birth also had cardiac malformations including atrioventricular-canal-type ventricular septal defects, ostium primum atrial septal defects, and enlarged atria (data not shown). All mutants specifically evaluated for a cardiac phenotype (*n* = 10) had abnormal cardiac development.

**Figure 2:**
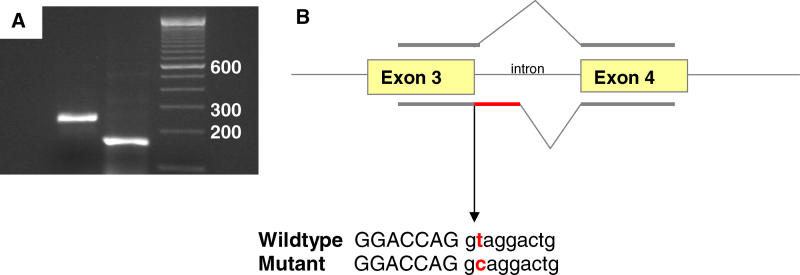
The ENU-Induced *lil* Mutation in *Fog2* is a Splice Site Mutation (A) RT-PCR revealed a lengthened transcript in mice with the *lil* phenotype: the first band is a *lil* mouse, and the second band is a control mouse. (B) Sequencing *Fog2* revealed a splice site mutation that causes the insertion of 85 bp of intronic sequence in the mutant mouse. This results in a premature stop codon prior to zinc finger transcription.

Examination of the 3-Mb region between *D15Mit85* and *D15Mit5* in DNA sequence databases revealed three predicted genes and four known genes, including *Fog2*. Targeted mutations of *Fog2* have cardiac defects strikingly similar to those we identified in *lil* mutant mice, including atrioventricular canal defects, thinned myocardium, and absent coronary vasculature [16]. RT-PCR amplification of the proximal portion of *Fog2* revealed longer transcripts in the *lil* mutant than in the wild-type embryos (Figure 2A). Sequencing of the mutant transcript revealed a point mutation (from thymine to cytosine) 2 bp after position 301, which is in the splice donor site at the end of the third exon. This mutation causes a splicing defect that results in the insertion of 85 bp of intronic sequence into the mutant transcript, and introduces a stop codon that generates a severely truncated protein product (Figure 2B). Heterozygous *lil* mutant mice were crossed with a *Fog2* ^+/−^ (null allele) mutant generated by gene targeting [16]. Doubly heterozygous mice had an embryonic lethal phenotype; this failure to complement proves that *lil* is a mutation in *Fog2*. The variable phenotype of *lil* mice (relative to that found for the *Fog2* null mutant) is likely due to the generation of a low level of normal transcript despite the presence of the splice site mutation.

### Lung and Diaphragm Development in the *Fog2* Mutant Mouse

Experiments were conducted to evaluate the role of *Fog2* in pulmonary and diaphragmatic development. The pulmonary phenotype is characterized by diffuse hypoplasia and specific loss of the accessory lobe and a portion of the right middle lobe.

**Figure 3:**
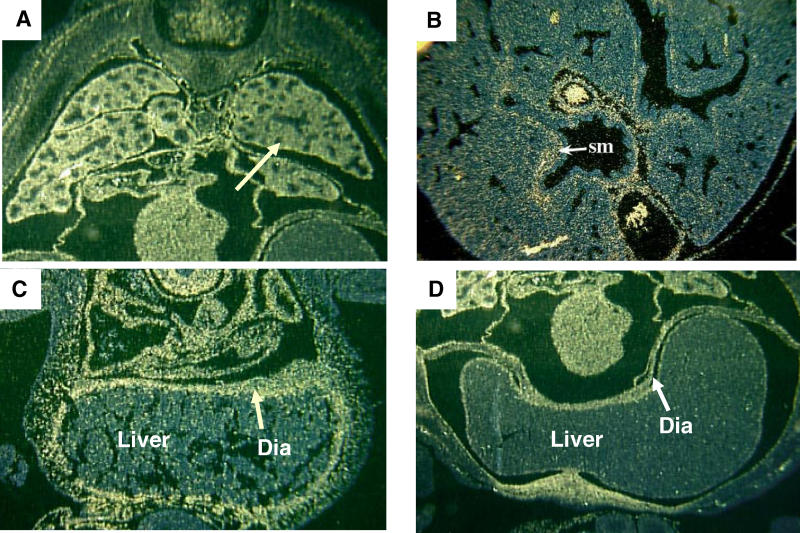
*Fog2* Is Expressed in the Developing Lung and Diaphragm *Fog2* is expressed in the diffuse pulmonary mesenchyme at E13.5 (A) (arrow shows mesenchyme) and is restricted to the bronchial and vascular smooth muscle (sm) at E16.5 (B). *Fog2* is expressed diffusely in the developing diaphragm (Dia) both prior to (E11.5) (C) and after muscularization (E13.5) (D).

**Figure 4:**
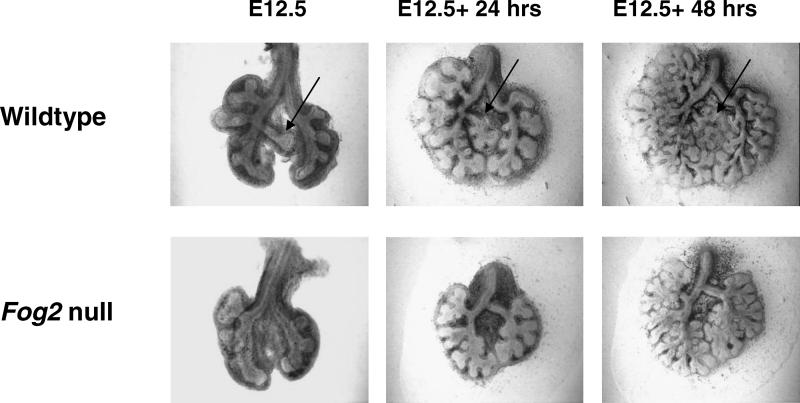
*Fog2* Is Necessary for Primary Lung Development *Fog2* null lungs removed prior to diaphragmatic muscularization and grown in vitro show no accessory lobe development. Accessory lobe is labeled with black arrow in control littermate lungs.

It is well established that abnormalities in either diaphragmatic development or fetal breathing can result in a secondary pulmonary hypoplasia, although loss of normal structure has never been documented in this context [17,18]. *Fog2* is expressed diffusely in the pulmonary mesenchyme during the period of branching morphogenesis, while later expression is restricted to the smooth muscles of airways and pulmonary vessels (Figure 3). This, and the observation that lungs appeared small on transverse sections evaluated prior to diaphragmatic muscularization or function, suggests that the pulmonary hypoplasia occurs independently of the diaphragmatic defect. To test this hypothesis, lungs were dissected from *Fog2* ^−/−^ mice and littermate controls before the onset of fetal diaphragmatic motion. *Fog2* ^−/−^ mice were used for this experiment, as we wanted to avoid potential phenotypic variance from the *lil* hypomorphic mutation. Lungs dissected at E12 from *Fog2* ^−/−^ embryos were smaller in size and lacked the development of an accessory lobe. In 11 viable *Fog2^−^* ^/−^ lung culture explants, there was never development of an accessory lobe, and the weights of mutant lungs cultured for 5 d were significantly lower than those of littermate controls (Figure 4). These data demonstrate that the pulmonary hypoplasia in *Fog2* mutant mice is a primary defect.

Branching in the unaffected lobes appeared to be delayed by 6–12 h, but all mutants developed an intricate branching pattern in the unaffected lobes after culture for 5 d.

**Figure 5:**
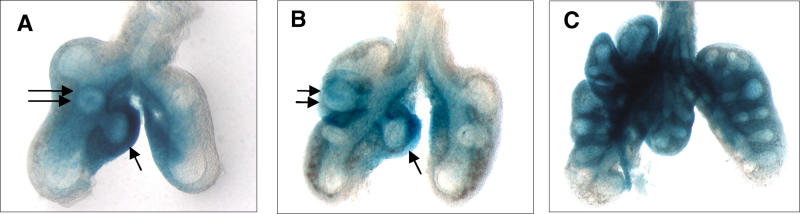
*Fog2* Expression in Embryonic Non-Mutant Lungs In embryonic non-mutant lungs, *Fog2* is most highly expressed at the tips of the accessory (single arrows) and right middle lobes (double arrows) at E11.25 (A) and E11.75 (B), while expression is diffuse by E12.75 (C).

Because the *Fog2* phenotype is striking for specific lobar loss, the spatial pattern of *Fog2* expression was evaluated in normal embryos during the period of early lobar establishment to determine whether *Fog2* expression is specifically different at these lobar buds. Expression was evaluated in mice carrying a *lacZ* gene incorporated into the *Fog2* locus (S. Tevosian, unpublished data). In nine mice examined at E11.5, all lungs showed a specific enhancement of *Fog2* expression in the mesenchyme surrounding the accessory bud and the right middle lobe bud, which are the lobes that do not develop normally in *Fog2* mutant mice (Figure 5). By E12.5, expression was diffuse in the pulmonary mesenchyme, as was seen previously with in situ hybridization on tissue sections.

**Figure 6:**
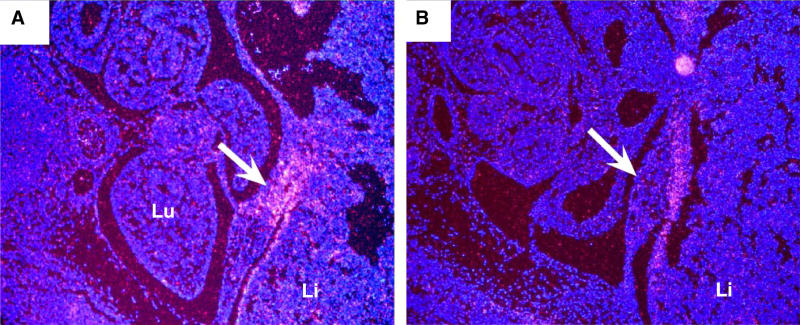
*HGF* Patterning Is Abnormal in *Fog2* Null Mice In situ hybridization of *HGF* in E12.5 wild-type (A) and *Fog2* ^−/−^ (B) embryos demonstrates decreased expression in the region where the PPF meets the membranous diaphragm. Li, liver; Lu, lung.

Diaphragms from *Fog2 lil* mice show an intact membrane with a defect in muscular patterning (see Figure 1B). The membranous portion of the diaphragm is populated by a migratory population of muscle precursor cells, much like the limbs [19,20]. Mice with defects in genes known to be important for the control of this process have intact but amuscular diaphragms [17,21,22]. *Hepatocyte growth factor/Scatter factor* *(HGF)* is one potential candidate responsible for the guidance of muscle precursors to the membranous diaphragm. It has been shown that *HGF* is expressed along this anatomic pathway [23], and mice with absence of the *HGF* receptor *c-Met* fail to form muscularized pleuroperitoneal folds (PPFs), and thus have amuscular diaphragms [24,25]. *Fog2* is expressed diffusely in the early amuscular diaphragm at E11.5 as well as in the later muscularized diaphragm (see Figure 3C and 3D). *Pax3* and *MyoD,* transcription factors required for appropriate migration and determination of myogenic precursors, were detected in the PPFs of *Fog2 lil* mice (data not shown). However, in situ expression analysis demonstrated that the expression of *HGF* in the region where this structure meets the membranous diaphragm was markedly reduced in *Fog2* mutant mice (Figure 6). We hypothesize that *Fog2* is required (either directly or indirectly) for the induction of *HGF* in the developing diaphragm, and dysregulation of *HGF* patterning along the path of muscle precursor cell migration between the PPF and the diaphragm accounts for the abnormal phenotype in these mice.

### *FOG2* Mutation in a Patient with Diaphragm and Lung Abnormalities

*FOG2* sequence analysis was performed on autopsy tissue from 30 of 32 deceased children with an anatomic diagnosis of diaphragmatic defect evaluated at the Children's Hospital in Boston, Massachusetts, between 1993 and 2003. Autopsy reports were reviewed to determine the specific diagnoses. Of these 30 cases, 17 (57%) had Bochdalek CDH, two (7%) had diaphragmatic agenesis, seven (23%) had diaphragmatic eventration/muscularization defects (without Bochdalek CDH), and four (13%) had Bochdalek hernia of one hemidiaphragm and eventration of the other. Pulmonary hypoplasia was assessed using lung/body weight ratio and radial alveolar counts [26]. The material available for review included written reports and histologic slides in all cases and gross kodachromes in a subset of cases.

One child carried a highly significant *FOG2* sequence change. The patient was a full-term 3,500-g baby girl who developed severe respiratory failure at birth and died after 5 h of resuscitation. Antemortem radiographs showed opacified lung fields and possible bowel in the chest. The patient's clinical diagnosis was CDH.

Review of autopsy material revealed severe bilateral pulmonary hypoplasia (combined lung weight, 11.1 g; expected for body length/weight, 46.8 ± 26.2 g; [27]), most markedly involving the left lung. The lung/body weight ratio was 0.0037 (expected > 0.010) [28]. There were a reduced number of bronchial generations, and the radial alveolar count was 2–3 (expected = 5) [29]. There were incomplete lung fissures bilaterally. A deep posterior diaphragmatic eventration was present on the left side. Additionally, two muscularized ligamentous bands resembling diaphragmatic remnants encircled the left lobe of the liver, creating an abnormal fissured liver contour. Away from the eventration, the diaphragm appeared well muscularized, measuring 0.3 cm in thickness. A complete autopsy revealed no other malformations; the heart was determined to be grossly normal and was donated for valve harvest.

**Figure 7:**
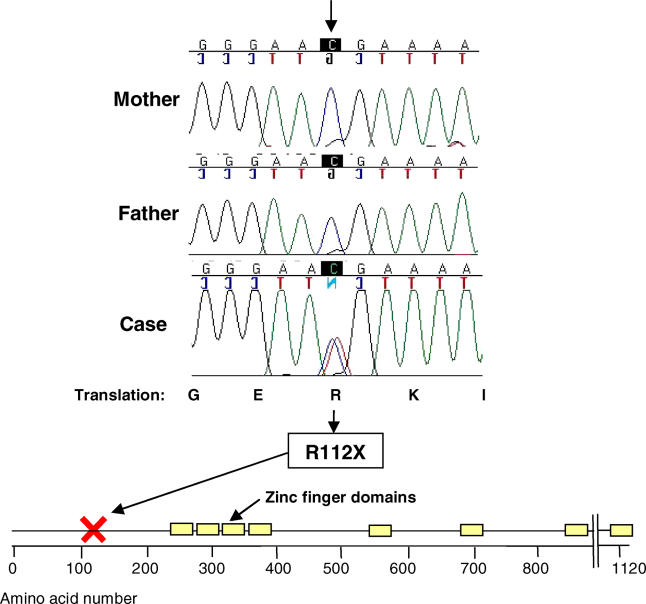
*FOG2* Mutation in a Patient with Diaphragm and Lung Abnormalities Sequencing revealed a de novo heterozygote nonsense mutation in a patient who died at birth with severe pulmonary hypoplasia and a posterior deep diaphragmatic eventration. She was clinically diagnosed with CDH. This nonsense mutation occurs prior to the functional zinc finger domains.

Sequence analysis revealed a cytosine to thymine heterozygous change in exon 4 that changes the 112th amino acid from arginine to a stop codon. This mutation produces a severely truncated peptide that does not contain zinc finger domains (Figure 7). This base change was not present in the analysis of DNA from 400 normal adults. To assess the likelihood that the mutation was causal for the developmental phenotype, we examined both parents. Paternity was confirmed, and sequence analysis revealed that the neither parent carried this mutation, proving that the patient had a de novo mutation in *FOG2* (Figure 7).

##  Discussion

Congenital diaphragmatic defects are a heterogeneous group of disorders of unknown etiology. The defects that present in the pre- or perinatal period include Bochdalek hernia, diaphragmatic aplasia, and various degrees of muscularization defects or eventrations. Different types of defects occur in the same patients or in siblings, suggesting these represent variable expression of the same underlying pathogenesis [30,31]. Clinical differentiation between these defects may be very difficult, as the residual membranous diaphragm of a muscularization defect is thin and may not be easily visible on prenatal ultrasound or postnatal chest radiographs [32]. Although diaphragmatic muscularization defects were historically considered to be predictive of a good outcome, there have been inadequate population-based studies that include fetal or neonatal cases and autopsy diagnoses to make this conclusion definitive. In fact, the series of patients we report here and the published literature indicate that an eventration defect may be associated with displacement of abdominal contents and also with severe pulmonary hypoplasia and respiratory insufficiency [33,34].

Numerous chromosome abnormalities have been found in association with congenital diaphragm abnormalities [12,35]. Human *FOG2* maps to Chromosome 8q23.1, and, importantly, several patients with diaphragm defects and rearrangements involving this locus have been reported. Specifically, there are three unrelated CDH patients with cytogenetically balanced translocations at or near the *FOG2* locus [36,37]. Additionally, two patients with deletions apparently encompassing the *FOG2* locus have died from multiple congenital anomalies including CDH [38–40]. Inactivation of this gene due to chromosomal rearrangement or deletion would result in a heterozygous null mutation similar to that found in the patient we report.

Because the *FOG2* mutation we report is de novo and the phenotypes of the pulmonary and diaphragmatic defects are similar between mouse and human, we suggest that this mutation in *FOG2* is the first reported cause of a human developmental diaphragmatic and pulmonary defect. In contrast to the affected child, mice heterozygous for a null mutation of *Fog2* appear normal. However, there is ample precedent for the observation that haploinsufficiency of a gene with developmental functions is much less well tolerated in humans than mice [41].

It is unclear how the *Fog2* diaphragmatic defect relates to the more common Bochdalek CDH, as the pathogenic mechanisms for both are largely unknown. Muscle precursors destined to populate the diaphragm migrate from the lateral dermomyotome of cervical somites. Prior to migration onto the diaphragm, they populate the PPF, a wedge-shaped tissue that tapers medially from the lateral body wall to the esophageal mesentery and fuses ventrally with the septum transversum [42]. Muscle precursors reach the PPF by E11, where they proliferate, differentiate, and then migrate toward the dorsolateral costal, sternal costal, and crural regions of the developing diaphragm. Thus, a defect in PPF formation subsequently results in the abnormal formation of the diaphragm [43]. We have shown that the *Fog2* mutant does have an abnormal pattern of *HGF* expression in the region through which muscle precursor cells migrate onto the developing diaphragm. This finding may account for the abnormally patterned muscle that develops in the *Fog2* mutant diaphragm. Although *Pax3* and *MyoD* expression is detected in the PPF, a detailed analysis of transcription factors responsible for muscle precursor cell migration and differentiation will need to be completed both in the PPF and along the pathway of muscle precursor cell migration between the PPF and the membranous diaphragm. *Fog2* can interact with any of the *Gata* factors, *Gatas 1–6,* as well as other transcription factors such as *CoupTFII* [44,45]. It is known that a *Fog2*–*Gata4* interaction is critical for normal cardiac and gonadal development, but interacting factors in the lung and diaphragm have not yet been determined.

The severity of pulmonary hypoplasia in the patient we report was out of proportion to that of the diaphragm defect. Pulmonary hypoplasia is associated with abnormal diaphragmatic anatomy or function, and is known to occur as a secondary developmental defect in models of diaphragmatic dysfunction such as complete amuscularization [17] or phrenic nerve disruption [46]. It occurs in a surgical model of CDH in which a hernia is physically created in an in utero lamb [47,48]. However, the possibility that primary pulmonary developmental abnormalities occur with, rather than secondary to, diaphragmatic defects has been suggested by others based on a teratogenic model of CDH [49–51] and has long been suspected by clinicians who care for these patients. In addition, the high incidence of lobar abnormalities associated with CDH [52] supports the possibility that this disorder can be associated with a primary developmental pulmonary abnormality.

Our analysis of mice carrying mutations of *Fog2* proves that there is a primary defect in lung development that results in specific loss of the accessory lobe and partial loss of the right middle lobe. The specific lobar defects prompted us to evaluate *Fog2* expression at the time of early lobar budding. While *Fog2* expression is diffuse in the pulmonary mesenchyme after lobar structure is well established (E12.5), it is more focally expressed in the mesenchyme surrounding the right middle lobe and accessory buds as these lobes form. This matches the phenotype of right middle lobe and accessory lobe loss, and suggests that *Fog2* has a specific patterning role in establishment of these lobes. It is less clear whether loss of *Fog2* results in a global branching defect, as *Fog2* lungs appear to have a slight developmental delay, which could result from many causes. Cultured *Fog2* lungs do develop an intricate branching pattern in the unaffected lobes that appears similar in the pattern to wild-type lungs after 5 d in culture.

In this report, we show that a mutation of *Fog2* in the mouse causes the phenotype of abnormal diaphragmatic muscularization and primary pulmonary hypoplasia. We furthermore demonstrate that a mutation in this gene is associated with a lethal defect in lung and diaphragm development in a child. It is notable that, despite extensive analysis of *Fog2* biology and the generation of a *Fog2* knock-out mouse, its role in diaphragm and lung development was previously not recognized. It is only as a consequence of phenotype-driven analyses such as those we are pursuing that one has the opportunity to assay all of the potential molecular derangements that may result in human disease.

## Materials and Methods

### 

These investigations were conducted with approval of the institutional review board for Children's Hospital, Boston, and Brigham and Women's Hospital, Boston. Animal use was approved by the Center for Animal Resources and Comparative Medicine (Harvard Medical School).

#### Genetic mapping of the mouse mutation *lil.*

The *lil* mutation was identified as described in results. Wild-type FVB/N and C57BL/6J mice used for genetic crosses were obtained from the Jackson Laboratory (Bar Harbor, Maine, United States). Mice carrying a null mutation of *Fog2* generated by gene targeting [16] were the generous gift of Dr. Stuart Orkin.

#### Developmental analysis of mice.

Timed pregnancies were set up for collection of E11.5–E17.5 embryos. Embryos were fixed, dehydrated, and embedded in paraffin prior to sectioning. In older embryos, a median sternotomy was performed under microscopic guidance, and diaphragm, lungs, and heart were examined. The lungs and tracheobronchial tree were removed and weighed. Whole diaphragms were isolated from fixed thoracic tissue from E15.5 and E17.5 embryos. For lung explant culture, lungs were dissected from fresh embryos at E11.5 and E12.5 and placed on porous 24-mm (0.4-μ) polyester membranes floated in wells containing 2 ml of Dulbecco's modified Eagle's medium, nutrient mixture F-12 (11039–021, Gibco, San Diego, California, United States), supplemented with 10% fetal bovine serum, 0.3 mg/ml L-glutamine, 100 units/ml penicillin, 100 mcg/ml streptomycin, and 0.25 mcg/ml amphotericin B. Lung explants were cultured at 37 °C in 95% air/5% CO_2_ for up to 5 d. They were photographed daily with a dissecting microscope (MZ12.5, Leica, Wetzlar, Germany) equipped with a Leica DC500 digital camera.

Transgenic mice carrying the *lacZ* gene driven by the *Fog2* promoter have been developed by S. Tevosian. In these animals, the *lacZ* gene is incorporated (“knocked-in”) into the *Fog2* locus to allow β-galactosidase expression as a fusion protein in frame with the first 235 amino acids of the FOG2 protein. The *Fog2-lacZ* module is followed by an ires-eGFP cassette. This creates a null allele of *Fog2* gene. The *Fog2-LacZ*-eGFP construct was linearized with KspI and electroporated into the CJ7 ES cells. The correctly targeted clone was selected by the Southern blot analysis and injected into C57BL/6J blastocysts. *Fog2-lacZ*-eGFP animals were maintained on the mixed C57BL/6J/129 background. *lacZ* Expression in whole dissected embryonic lungs was analyzed by staining for β-galactosidase activity with X-gal after fixation for 30 min.

#### RT-PCR and sequence analysis in the mouse.

RNA was extracted by standard techniques from thoracic embryonic tissue. RT-PCR was performed using six primer sets designed to cover the *Fog2* gene. RT-PCR was repeated with radiolabeled primers to amplify an abnormally spliced region of the gene (Table S1), and the product was run on a denaturing sequencing gel according to standard techniques. The RT-PCR product was cloned into pCR2.1 vector using TOPO TA Cloning Kit (Invitrogen, Carlsbad, California, United States) and sequenced using gene-specific primers. Sequence analysis was done using Sequencher 4.1 (Gene Codes, Ann Arbor, Michigan, United States).

#### In situ hybridization.

After dehydration and embedding in paraffin wax, 10-μ sections were subjected to radioactive in situ hybridization as described [53]. Probes labeled with 35S were prepared by run-off transcription of linearized plasmid templates and hybridized to tissue sections. Nuclei were counterstained with Hoescht 33258, and signal was imaged using fluorescent and darkfield microscopy.

#### Human DNA extraction and sequence analysis.

DNA was isolated from paraffin blocks by phenol-chloroform extraction [54,55], and from frozen tissues by standard techniques. Primers were designed to amplify *FOG2* coding exons plus 50 bp of flanking upstream and downstream sequence. PCR amplification and sequencing were performed by standard methods. Primer sequences used are listed in Table S2. Sequence analysis was done with Sequencher 4.1 (Gene Codes).

DNA from the parents of one autopsy patient was extracted from fresh blood samples. A second set of blood samples was sent to an outside CLIA-certified laboratory for DNA extraction, PCR, sequencing, and analysis. Paternity testing was performed by the outside laboratory using a standard panel of markers. SNP genotyping was done using Harvard Partners Center for Genetics and Genomics genotyping core facility (Cambridge, Massachusetts, United States).

## Supporting Information

Table S1 Primers for RT-PCR (Mouse): Amplification of Abnormal Transcript in *Fog2* Mutant *(lil)* Mice (26 KB DOC)

Table S2 Human Primers for Amplification of *FOG2* Coding Sequence from Genomic DNA (46 KB DOC)

## Abbreviations

CDH: congenital diaphragmatic hernia
E[number]: embryonic day [number]
ENU: N-ethyl-N-nitrosourea
*HGF*: *Hepatocyte growth factor/Scatter factor*
*lil*: *little lung*
PPF: pleuroperitoneal fold
